# Association Between Marijuana Use and Cardiovascular Disease in US Adults

**DOI:** 10.7759/cureus.11868

**Published:** 2020-12-03

**Authors:** Dhaval Jivanji, Maverick Mangosing, Sean P Mahoney, Grettel Castro, Juan Zevallos, Juan Lozano

**Affiliations:** 1 Urology, Florida International University, Herbert Wertheim College of Medicine, Miami, USA; 2 Internal Medicine, Florida International University, Herbert Wertheim College of Medicine, Miami, USA; 3 Medical and Population Health Sciences Research, Florida International University, Herbert Wertheim College of Medicine, Miami, USA; 4 Epidemiology and Public Health, Florida International University, Herbert Wertheim College of Medicine, Miami, USA; 5 Miscellaneous, Florida International University, Herbert Wertheim College of Medicine, Miami, USA

**Keywords:** cannabis, cardiovascular disease, marijuana, prevalence

## Abstract

Introduction

The prevalence of marijuana use has increased by about 16% since 2006, translating to approximately 200 million people worldwide. Being so widely used, long-term effects of marijuana use on cardiovascular health are largely unknown. Previous studies have had conflicting results, either showing marijuana use having a negative impact or no significant impact on cardiovascular health. This study aims to add evidence regarding the impact marijuana use has on the prevalence of cardiovascular disease.

Methods

This retrospective study was conducted using the Behavioral Risk Factor Surveillance System (BRFSS) database. Patients who completed the questionnaire and answered all questions in relation to marijuana use and the diagnosis of cardiovascular disease in 2017 were a part of this study. Subjects were excluded if they were children (<18 years old) or had missing data for marijuana use or cardiovascular disease. Age, gender, race/ethnicity, body mass index (BMI), income, exercise, tobacco use, alcohol use, and depression were all considered as potential confounders. Bivariate analysis was conducted to find an initial association between marijuana use and cardiovascular disease, which was followed by a multivariate regression analysis to adjust for confounders. Odds ratios and 95% confidence intervals were calculated.

Results

A total of 56,742 subjects were included in the analysis. The unadjusted bivariate analysis showed a reduced prevalence of cardiovascular disease in individuals using marijuana (OR 0.65, 95%CI [0.50-0.84]). After adjustment with all additional variables, an adjusted model showed a similar odds ratio, but statistical significance of the association was lost (OR 0.74, 95%CI [0.54-1.01]).

Discussion

A systematic review by Ravi et al in 2018, which looked at marijuana use, cardiovascular risk factors, and clinical outcomes concluded that there was insufficient data to make conclusions regarding the effect of marijuana use and negative long-term cardiovascular effects. Our study lends support to the notion that marijuana use does not have an association with cardiovascular disease. A limitation in our study was that there was missing data from the BRFSS questionnaire due to participants not fully answering all questions concerning cardiovascular disease and marijuana use. This decreased our sample size from 67,974 to 56,742 subjects. The missing participants led to a decrease in the power of our odds ratio, which may have impacted statistical significance of our results.

Conclusion

Although previous literature has shown that marijuana use has a negative impact on cardiovascular health, our study suggests that users and non-users of marijuana did not have an association with the prevalence of cardiovascular disease. Varying levels of support within the literature highlights the need for further research of this association.

## Introduction

Marijuana is a widely known psychoactive flowering plant that is being used both recreationally and for medicinal purposes. The prevalence of marijuana use has increased by about 16% since 2006, translating to approximately 200 million people worldwide [1]. With this substance use being increasingly prevalent, especially within the scope of medical therapeutics, it is crucial to have evidence about the long-term effects of marijuana use on cardiovascular diseases, as it has been shown in multiple studies to have an effect on blood pressure, heart function, and sympathetic drive [1-4]. Currently, there is very mixed evidence about the impact of marijuana use and subsequent cardiovascular issues. Some note that marijuana use can acutely increase blood pressure for two to five hours as well as increase cardiac output by more than 30% [2]. There are also suggestions that the onset of myocardial infarction (MI) increases in the first 60 minutes after marijuana consumption [5]. Furthermore, in daily marijuana users, the annual risk of MI increases from 1.5% to 3.0% per year. Through both human and animal trials, it is suggested that this may be due to coronary artery vasospasm induced by marijuana use [3]. Lastly, a systematic review published in February of 2018 concluded that while there have been studies done that have found associations between cardiovascular risk factors and marijuana use, there is insufficient evidence to conclude anything regarding this link [4]. With such varied results from multiple peer-reviewed articles, there is not a clear picture of how marijuana use impacts cardiovascular health. Through this project, we hope to add evidence to the association between marijuana use and cardiovascular disease. This aims to pinpoint any demographic characteristics or combinations of risk factors that positively or negatively impact patient health. 

## Materials and methods

This retrospective cross-sectional study was conducted using data from the 2017 Behavioral Risk Factor Surveillance System (BRFSS) annual report. The BRFSS collects data via telephone surveys given in all 50 states and three US territories. It used a random sample, as telephone numbers were gathered via a random number generator. The initial sample was filtered to include US adults (above 18 years) from states that asked each subject a relevant question in the survey regarding marijuana use. States that are part of this study included: Alaska, California, Georgia, Idaho, Minnesota, New Hampshire, South Carolina, Tennessee, and Wyoming. These were the only states included as the survey questions regarding marijuana use were asked exclusively in these nine states. Subjects were excluded from this sample if they had any incomplete data regarding marijuana use, cardiovascular disease, or both. 

The primary exposure for the study was marijuana use. Within the database marijuana use was assessed by asking: “During the past 30 days, on how many days did you use marijuana or cannabis?”. Data were summarized into two categories: “yes” if a subject answered any number > 0, or “no” if the subject answered 0. Our primary outcome was the prevalence of cardiovascular disease. Questions associated with the prevalence of cardiovascular disease included: “Has a doctor, nurse, or other health professional ever told you that you had a heart attack also called a myocardial infarction?”, “(Ever told) you had angina or coronary heart disease?”, and “(Ever told) you had a stroke?”. Subjects that answered “no” to all 3 questions were designated as not having cardiovascular disease, while those answering “yes” to any of them were considered otherwise. Additional variables that factored as potential covariates included: age (<65 years old, ≥ 65 years old), gender (Male, Female), race/ethnicity (White only, Black/African American only, Asian only, Other, Multiracial, Hispanic), body mass index (BMI) (underweight [BMI<18.5], normal weight [BMI 18.5-24.99], overweight [BMI 25-29.99], obese [BMI ≥30]), annual household income (< $25,000, $25,000-49,999, $50,000-74,999, >$75,000), exercise in past 30 days (yes/no), tobacco use defined as 100 or more cigarettes over entire life (smoker/non-smoker), alcohol use in the past 30 days (yes/no), and medically diagnosed depression (yes/no). 

All analysis was conducted using STATA version 14 (StataCorp., College Station, TX). First, chi-square tests were performed to analyze the distribution of baseline characteristics (i.e., age, gender, race/ethnicity, BMI, income, exercise, tobacco use, alcohol use, depression) in relation to marijuana use. The associations between marijuana use and cardiovascular disease, as well as among other baseline features and cardiovascular disease, were then assessed using bivariate analysis. A multivariate logistic regression was conducted to determine and control for potential covariates on the association between the primary exposure and the outcome. Odds ratios and 95% confidence intervals were computed to quantitatively express any association. Lastly, because many eligible participants were ultimately excluded due to missing data, we conducted best-case and worst-case scenarios for the missing subjects, placing all missing subjects either in the non-marijuana user or marijuana user categories, respectively. Furthermore, baseline features between subjects in the study and missing subjects were also compared.

## Results

**Figure 1:**
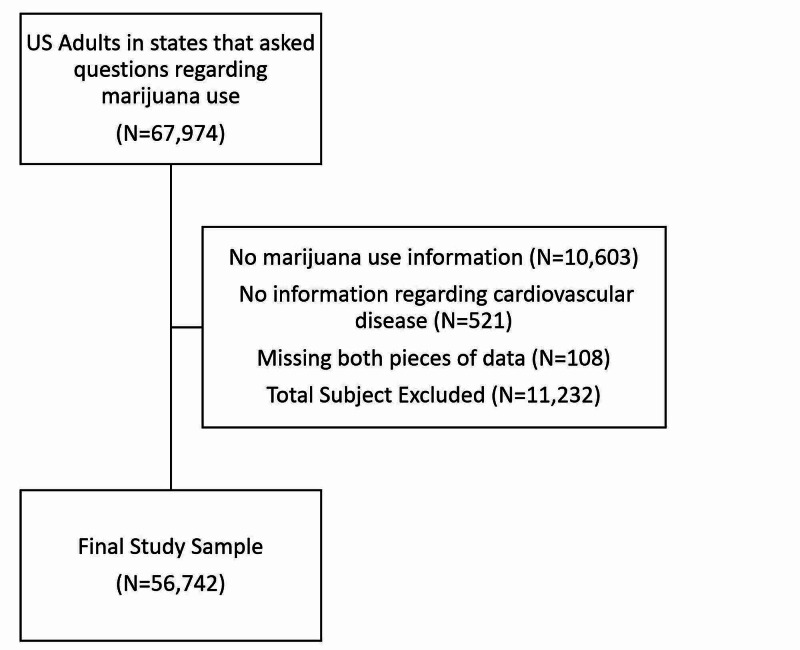
Selection of final study sample

The 2017 BRFSS annual report included 67,974 participants. Subjects were excluded from this initial sample if they had any incomplete data regarding marijuana use cardiovascular disease, or both. In total, 11,232 subjects were excluded applying these criteria, leaving a final sample size of 56,742 individuals (Figure 1). 

Among our sample, the overall prevalence of marijuana use was calculated as 3,412 out of 56,742 subjects (6.0%) (Table 2). When analyzing the baseline characteristics of our sample, all but 2 of our covariates demonstrated a statistically significant difference between those who used marijuana versus those who did not (Table 1). Younger age (<65 years old) was more common in marijuana users (93.2%) versus non-marijuana users (77.1%). Marijuana users were also more commonly male (63.1%) and reported exercising more (80.7%). The frequency of tobacco use was significantly higher in marijuana users (58.6%) versus non-marijuana users (35.5%). Furthermore, marijuana users tended to answer yes to screening questions on depression (31.1%) versus non-marijuana users (17.4%). When examining overweight and obese individuals via body mass index, 32% of overweight individuals and 20.8% of obese individuals used marijuana. A higher percentage of marijuana users consumed alcohol (40.0%) when compared to non-marijuana users (13.1%). Both the variables of race/ethnicity and income status did not show a statistically significant difference (p<0.001) between the two groups. 

**Table 1:** Baseline characteristics of the sample of US adults and their marijuana use, according to completion of the BRFSS questionnaire BRFSS, Behavioral Risk Factor Surveillance System

Characteristics	Marijuana Use				p-value	
	No		Yes			
	N	%	N	%		
Age						
<65 years old	33,197	77.1	2,989	93.2	<0.001	
65 years or older	19,548	22.9	409	6.8		
Gender						
Male	23,090	46.4	2,147	63.1	<0.001	
Female	30224	53.6	1,264	36.9		
Race/Ethnicity						
White	41,205	55.7	2,403	55.8	0.0145	
Black/African American	4723	10.7	292	12.8		
Asian	902	9.1	57	6.6		
Other	1,131	1.5	171	2.2		
Multiracial	813	1.4	122	2.3		
Hispanic	3,547	21.6	302	20.3		
BMI						
Underweight (<18.5)	752	1.92	92	2.6	<0.001	
Normal weight (18.5-24.99)	15,109	31.9	1,341	44.6		
Overweight (25-29.99)	18,123	36.2	1,147	32.0		
Obese (≥30)	15,705	30	771	20.8		
Income						
< 25,000	11,408	27.7	1,026	28.8	0.4345	
25,000-49,999	11,125	22	724	19.7		
50,000-74,999	7,410	14.6	469	14.5		
≥75,000	15,558	35.7	875	37.0		
Exercise						
+	38,537	74.9	2,624	80.7	<0.001	
0	14,419	25.1	770	19.3		
Tobacco use						
+	21,908	35.5	2,277	58.6	<0.001	
0	31,134	64.5	1,125	41.4		
Alcohol use						
+	5,964	13.1	1,345	40.0	<0.001	
0	46,542	86.9	1,994	60.0		
Depression						
+	10,163	17.4	1,180	31.1	<0.001	
0	42,953	82.6	2,204	68.9		

Table 2 shows the prevalence of cardiovascular disease among individuals within our sample. Overall, 11.2% (N = 6,353) of the sample reported some form of cardiovascular disease. Relating it to the exposure, 5.5% of subjects that use marijuana reported having cardiovascular disease, as compared to 8.2% among non-marijuana users. As expected, older individuals (>65 years old) showed a higher prevalence (19.9% vs. 4.7%) of cardiovascular disease when compared to their younger counterparts. The overall prevalence of cardiovascular disease was highest in multiracial and other race groups (10.6% and 10.9%, respectively), followed closely by that in whites (9.8%). Other baseline characteristics showed the expected association with cardiovascular disease. Those with higher BMIs, lower income, no exercise, tobacco use, alcohol use, and depression all had a significantly higher prevalence of cardiovascular disease (p < 0.001). 

**Table 2:** Baseline characteristics of the sample of US adults and cardiovascular disease, according to completion of the BRFSS questionnaire BRFSS, Behavioral Risk Factor Surveillance System

Characteristics	Cardiovascular Disease				p-value	
	No		Yes			
	N	%	N	%	N	
Marijuana Use						
0	47,259	91.8	6,071	8.2	<0.001	
+	3,130	94.5	282	5.5		
Age						
<65 years old	34,016	95.3	2,170	4.7	<0.001	
65 years or older	15,827	80.1	4,130	19.9		
Gender						
Male	21,899	90.6	3,338	9.4	<0.001	
Female	28,476	93.4	3,012	6.6		
Race/Ethnicity						
White only	38,605	90.3	5,003	9.8	<0.001	
Black/African American only	4,376	91.8	639	8.2		
Asian only	934	96.8	25	3.2		
Other	1,125	89.1	177	10.9		
Multiracial	804	89.4	131	10.6		
Hispanic	3,636	95.5	213	4.5		
BMI						
Underweight	730	94.2	114	5.8	<0.001	
Normal weight	14,949	94.1	1,501	5.9		
Overweight	17,099	91.5	2,171	8.5		
Obese	14,216	89.6	2,260	10.4		
Income						
< 25,000	10,235	87.9	2,199	12.1	<0.001	
25,000-49,999	10,463	91.5	1,386	8.5		
50,000-74,999	7,121	93.5	758	6.5		
At least 75,000	15,453	95.1	980	4.9		
Exercise						
+	37,313	93.3	3,848	6.7	<0.001	
0	12,733	88.3	2,456	11.7		
Tobacco use						
+	20,381	87.5	3,804	12.5	<0.001	
0	29,745	94.8	2,514	5.2		
Alcohol use						
+	6,936	95.5	373	4.5	<0.001	
0	42,652	91.4	5,884	8.6		
Depression						
+	9,537	86.6	1,806	13.4	<0.001	
0	40,649	93.3	4,508	6.7		

The unadjusted odds ratio of cardiovascular disease with marijuana use was statistically significant 0.65 (95% CI: 0.50-0.84) compared to those who did not use marijuana (Table 3). When adjusting for age, gender, race/ethnicity, BMI, income, exercise, tobacco use, alcohol use, and depression, the odds ratio was 0.74 (95% CI: 0.54-1.01), which was not statistically significant. The other variables that were strongly associated with cardiovascular disease after adjustment were age (>65 years old), income (<$25,000 per year), and depression. These variables each had an odds ratio > 2 (Table 3). 

**Table 3:** Unadjusted and Adjusted associations between marijuana use and cardiovascular disease in US adults who completed the BRFSS questionnaire BRFSS, Behavioral Risk Factor Surveillance System

Characteristics	Unadjusted		Adjusted		
	OR (95% CI)	p-value	OR (95% CI)	p-value	
Marijuana Use	0.65 (0.50-0.84)	0.001	0.74 (0.54-1.01)	0.059	
Age > 65 years	5.03 (4.44-5.70)	<0.001	4.36 (3.70-5.13)	<0.001	
Female Sex	0.68 (0.60-0.76)	<0.001	0.50 (0.42-0.58)	<0.001	
Race/Ethnicity					
White only	Reference	Reference	Reference	Reference	
Black/African American only	0.83 (0.69-0.99)	0.04	0.78 (0.61-1.01)	0.064	
Asian only	0.31 (0.18-0.52)	<0.001	0.77 (0.44-1.36)	0.372	
Other	1.14 (0.77-1.67)	0.519	1.02 (0.63-1.65)	0.139	
Multiracial	1.10 (0.72-1.69)	0.645	1.03 (0.60-1.76)	0.927	
Hispanic	0.44 (0.35-0.55)	<0.001	0.57 (0.44-0.76)	<0.001	
BMI					
Underweight	0.98 (0.66-1.46)	0.914	0.80 (0.48-1.33)	0.388	
Normal weight	Reference	Reference	Reference	Reference	
Overweight	1.49 (1.26-1.75)	<0.001	1.38 (1.15-1.67)	0.001	
Obese	1.85 (1.57-2.18)	<0.001	1.63 (1.35-1.97)	<0.001	
Income					
< 25,000	2.67 (2.23-3.18)	<0.001	2.44 (1.99-3.00)	<0.001	
25,000-49,999	1.79 (1.48-2.18)	<0.001	1.42 (1.14-1.77)	0.002	
50,000-74,999	1.35 (1.07-1.69)	0.01	1.16 (0.90-1.48)	0.246	
At least 75,000	Reference	Reference	Reference	Reference	
No Exercise in Past 30 Days	1.85 (1.64-2.09)	<0.001	1.42 (1.22-1.66)	<0.001	
Tobacco use	2.61 (2.31-2.95)	<0.001	1.77 (1.51-2.07)	<0.001	
Alcohol use	0.50 (0.39-0.63)	<0.001	0.66 (0.50-0.88)	0.005	
Depression	2.17 (1.90-2.49)	<0.001	2.19 (1.83-2.62)	<0.001	

Additional analysis was performed to identify any differences in the baseline characteristics between the individuals that had to be excluded due to missing data and those included. Notably, 87.1% of the excluded participants were under 65 years old, compared to 78.7% for the participants in the study. When analyzing differences in race, there was a higher proportion of Blacks (11.9% vs. 10.9%), Hispanics (24.3% vs. 21.4%), and Asians (12.7% vs. 8.8%) in the excluded group versus included group; however, there was a lower proportion of Whites (47.4% vs. 55.7%). Sex, BMI, income, exercise, tobacco use, and depression all showed similar distributions between each other. 

Lastly, a sensitivity analysis was done to check if the association between marijuana use and cardiovascular disease changed assuming that patients with missing information were all marijuana-users (worst-case scenario) or all non-users (best-case scenario). The worst-case scenario was statistically significant (OR: 0.78, 95% CI: 0.62-0.97), compared to the best-case scenario which was not (OR: 0.77, 95% CI: 0.57-1.06). 

## Discussion

Marijuana use has been shown to acutely increase cardiac output, affect blood pressure, and sympathetic drive [1-4]. As marijuana use is becoming more accessible through increased legalization, its long-term effects on users are largely unknown. Studies looking at marijuana use, and cardiovascular disease have shown mixed results [3,6,7]; therefore, additional studies looking at such association are warranted. 

The primary aim of this study was to evaluate the relationship between marijuana use and cardiovascular disease. After controlling for several confounding variables, we found that there was a decrease in the prevalence of cardiovascular events with marijuana use (OR: 0.74, 95%CI: 0.54,1.01), although it was not a statistically significant result.

A more comprehensive look at similar studies was provided by a systematic review by Ravi et al in 2018, which looked at marijuana use, cardiovascular risk factors, and clinical outcomes [4]. The study looked at a total of 24 separate studies, investigating both cardiovascular risk factors as well as clinical outcomes. Within the review, six of the studies revealed potential metabolic benefits in individuals using marijuana. Such information can be translated back to our study’s findings which could suggest an underlying physiological mechanism that could be cardioprotective. In looking at cardiovascular disease, one study found an increase in myocardial infarction risk in the first hour after smoking marijuana. Another three separate studies all found no association between marijuana use and the incidence of strokes. Finally, four more studies looked at marijuana use and all-cause cardiovascular mortality, but the results of the four studies varied. The overlying conclusion of the review showed insufficient data to make conclusions regarding the effect of marijuana use and negative long-term cardiovascular effects. In contrast, Rumalla et al. conducted a case-crossover study that found marijuana use to cause a 17% increase in the likelihood of hospitalization due to acute ischemic stroke (AIS) [8]. It was also found that the relative risk of individuals was highest among younger adults aged 25-34. This study adjusted for similar covariates to increase the accuracy of the analysis. Differences in findings compared to our study may stem from the fact that this study only focused on one specific form of cardiovascular disease, whereas our study took an umbrella approach to include several types of cardiovascular disease. Additionally, the sample gathered in this study focused on hospitalized patients rather than the general presence of cardiovascular disease. Nonetheless, this study reveals the negative impact that can be seen in people using marijuana. 

Since our data was extracted from BRFSS, a national database, gathering the initial dataset was quick and inexpensive. Using secondary data eliminated the need to develop any questionnaires or surveys to collect the data ourselves, which allowed for immediate statistical analysis of the sample generated. Additionally, the sample that was analyzed excluded any patient with missing data for both marijuana use and cardiovascular disease. This aimed to reduce any bias that may stem from these subjects, which could skew any association that may be present. Lastly, we made sure to adjust and control for several covariates (age, income, tobacco use, alcohol use, etc.) to make the most accurate analysis of our data. 

Our study had several limitations to note. As a whole, the study was conducted in a retrospective manner, which could affect the validity of the collection process and therefore impact the findings. Additionally, marijuana use was limited to the last 30 days before the completion of the survey. The only BRFSS survey question that gave information relevant to marijuana usage asked how many days the subject used marijuana in the last 30 days. There may have been marijuana users that did not use the drug in the last 30 days, or there may be very seldom users that happened to use it in that same time period. With a limited sample size, we did not break down levels of marijuana use, such as heavy users using marijuana >15 days, light users <15 days, etc. Therefore, not only was it not an exact process grouping marijuana users and non-marijuana users but within the marijuana users’ group, there were varying levels of usage. The sample size was inherently limited due to the fact that the BRFSS survey data regarding marijuana use was limited to only a subset of states. Moreover, the sensitive nature of this question could have impacted the accuracy of reporting.

Furthermore, an additional limitation we recognize is that chronic conditions such as hypertension and diabetes could increase the risk of developing cardiovascular disease. However, the primary aim of our study was to determine the prevalence of diagnosed CVD when surveyed. We do acknowledge the impact this could have had on our results, and therefore we believe further research should include these variables for a more robust analysis of this potential association. 

Another limitation was that there was missing data from the BRFSS questionnaire due to participants not fully answering all questions concerning cardiovascular disease and marijuana use. This decreased our sample size from 67,974 to 56,742 subjects. In addition, we recognize that there may have been a subset of respondents who answered "no" to the three questions used to designate subjects as having cardiovascular disease, which could affect the prevalence of our outcome. However, this could also be attributed to our data being gathered from self-reported surveys. 

Age, income, and depression were of importance as these variables had odds ratios >2 when linked to cardiovascular disease. As mentioned in the results, our sample had a higher proportion of the excluded data being from a younger subset of individuals. This could have affected prevalence of marijuana use as studies show that younger individuals have had higher odds of being marijuana users [9]. The other two variables, income and depression, showed a similar distribution between subjects included and excluded from the study. Previous studies have shown disparities between racial minority groups compared to whites concerning prevalence of marijuana use. Among these groups, black adults appear to have higher odds of weekly, monthly, and dependence on cannabis use [10]. This may have contributed to the increased proportion of black adults (<65) in the excluded sample compared to the included sample. 

Lastly, the answers gathered by subjects through the BRFSS questionnaire are self-reported. There is still a stigma against marijuana use in the United States, especially in the states included in the database that have not legalized it. Given this stigma and the perceived risk of admitting an unlawful behavior, subjects could have been affected by social desirability bias and therefore underreported their use of marijuana. This may have prompted subjects to inaccurately report that they did not use marijuana in the past 30 days. 

The link between marijuana use and cardiovascular disease in research is varied. Since our study lacks statistical significance, along with the limitations discussed, further research is implicated to determine if any association exists between marijuana use and cardiovascular disease. As the number of states legalizing marijuana use increases, studies with a similar process should be conducted. This could allow for a more accurate representation of the true prevalence of marijuana use, which would allow future studies to provide increased clinical utility of marijuana use and its impact on cardiovascular disease. Furthermore, because of the link between age and cardiovascular health, as well as the fact that younger age groups are more likely to use marijuana, a confounding variable becomes the age of the participant. As sample sizes grow larger, future studies may want to try to conduct a similar study, but in a specific age group to limit this covariate. Overall clinical significance of marijuana use cannot be fully elucidated given the fact that longitudinal data is still being gathered.

## Conclusions

In conclusion, our study found that there is no link to marijuana use and an increase in cardiovascular disease. Furthermore, there may be a link between marijuana use and lowered risk of cardiovascular disease (OR: 0.74, 95% CI: 0.54-1.01), but the data was not statistically significant when adjusting for confounding variables. This study does, however, implicate the need for future studies with other methods and/or larger sample sizes to provide more insight into this potential association. 
